# Lead versus lag-time trade-off variants: does it make any difference?

**DOI:** 10.1007/s10198-013-0505-0

**Published:** 2013-07-31

**Authors:** Federico Augustovski, Lucila Rey-Ares, Vilma Irazola, Mark Oppe, Nancy J. Devlin

**Affiliations:** 1 Institute of Clinical Effectiveness and Health Policy (IECS), Dr. Emilio Ravignani 2024, C1414CPV Buenos Aires, Argentina; 2 School of Public Health, University of Buenos Aires School of Medicine, Buenos Aires, Argentina; 3 Department of Health Policy and Management, Institute for Medical Technology Assessment, Erasmus University Rotterdam, Rotterdam, The Netherlands; 4 Office of Health Economics, London, UK

**Keywords:** Time trade-off, Lead-time TTO, Lag-time TTO, Worse than dead, EQ-5D-5L, Quality of life, I10, C93, D01

## Abstract

**Objectives:**

The traditional time trade-off (TTO) method has some problems in the valuation of health states considered worse than dead. The aim of our study is to compare two TTO variants that address this issue: lead-time and lag-time TTO.

**Methods:**

Quota sampling was undertaken in June 2011 in Buenos Aires as part of the EQ-5D-5L Multinational Pilot Study. Respondents were randomly assigned to one of the TTO variants with two blocks of five EQ-5D-5L health states. Tasks were administered using a web-based digital aid (EQ-VT) administered in a group interview.

**Results:**

A total of 387 participants were included [mean age 38.85 (SD: 13.97); 53.14 % females]. The mean observed values ranged from 0.44 (0.59) for state 21111 to 0.02 (0.76) for state 53555 in the lead-time group and between 0.53 (0.52) and 0.08 (0.76) in the lag-time group. There were no statistically significant differences in the values between TTO variants, except for a significant difference of 0.19 for state 33133. In both variants, marked peaks were observed around the value 0 across all states, with a higher percentage of 0 responses in the last state valued, suggesting ordering effects.

**Conclusions:**

No important differences were found between TTO variants regarding values for EQ-5D-5L health states, suggesting that they could be equivalent variants. However, differences between the two methods may have been obscured by other aspects of the study design affecting the characteristics of the data.

## Introduction

Time trade-off (TTO) is one of the most widely used methods for valuing health-related quality of life. Many of the EQ-5D value sets—generated in countries ranging from the UK [1] to the USA [2], Japan [3], the Netherlands [4], France [5], and Argentina [6]—were elicited from the general population using TTO. With TTO one finds the value for each health state (H*i*) by establishing the amount of time in full health (*x*) that is considered equivalent to a given amount of time in a poor health state (*t*). That value is calculated as (*x*/*t*). In EQ-5D valuation studies, *t* is set at 10 years.

Despite its widespread application, there are some problems with TTO, especially regarding the valuation of states that are considered ‘worse than dead’ (WTD) [7] because the trade-off procedure employed for WTD is different than the one used for states deemed better than dead (BTD). Accordingly, different TTO elicitation techniques are used for BTD and WTD. The TTO values for BTD are obtained by varying the years spent in full health (*x*), while the length of time spent in the health state (H*i*) that is to be evaluated is fixed (*t*). The procedure for WTD, in contrast, involves simultaneously changing both *x* and *t*.

Several researchers have noted the problems related to this approach. Above all, they question the assumption that both procedures produce utilities on the same scale, pointing out the possibility of obtaining extremely negative values for WTD [8, 9]. Such issues have far-reaching implications for the use of TTO values in health technology assessment and economic evaluations.

**Fig. 1:**
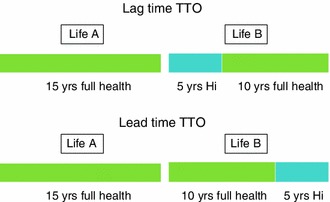
Lead-time and lag-time TTO variants

In 2006 Robinson and Spencer [10] proposed a solution to these problems: a new elicitation procedure capable of yielding values both above and below zero. The method involves simply adding some additional time in full health to the time available for trading (‘Life A’), as well as to the scenario comprising the state being valued (H*i*) (‘Life B’). In the latter, the time in full health *precedes* the scenario of illness presented in the TTO task, hence its description as a lead-time TTO; see Fig. 1. The objective of the task, as in conventional TTO, is to find a point of indifference between the two options. When the health state to be valued is considered better than dead, the point of indifference will be reached when the duration of Life A is longer than the period of years in full health offered in Life B. If the health state is considered worse than dead, at the point of indifference the duration of Life A will be shorter than the years in full health presented in Life B.

In lead-time TTO, the iterative trading process allows the participant to move between negative and positive values without being required explicitly to think about whether the state is worse or better than being dead. Unlike the conventional approach, lead-time TTO does not require a separate method of elicitation for these states. Thus, it avoids the ‘focusing effect’ that may arise with such deliberation. The question remains whether the resulting values <0 concur with the states judged as worse than being dead [11].

Devlin et al. [12] have shown that lead-time TTO is a feasible method for the valuation of health states in EQ-5D. They have provided evidence that this method may change the values for both BTD and WTD. Some questions regarding the length of time in full health to be presented in Life A and the adequate ratio of time in full health (lead-time) to time in H*i* presented in Life B have been explored [11]. Some of these issues warrant further investigation, however.

A related TTO variant called lag-time TTO has also been proposed. It places the additional time in full health *after* the health state to be valued instead of *before* it, as in lead-time TTO [7, 13] (Fig. 1). The method is therefore similar to that of lead-time TTO except for the temporal repositioning of time spent in poor health in Life B. Devlin et al. [11] investigated the difference between lead-time and lag-time TTO in the UK and also investigated the effect on values of offering varying amounts of time in full health relative to the duration of H*i*.

Some authors suggest that, disregarding potential problems with time preference and framing effects, the valuations should theoretically be the same for both lead- and lag-time TTO. Nonetheless, it is possible for the variants to differ in other respects (i.e., the two approaches differ in the placement of the poor health state relative to dead, so preferences regarding health at the end of life may lead to differences in values) [13].

The aim of this study is to understand how the temporal placement of the additional time in full health—either before (lead-time TTO) or after (lag-time TTO) the sub-optimal health state—influences the valuation of EQ-5D-5L health states.

## Methods

This study is part of the EQ-5D-5L Multinational Pilot Study conducted in the UK, the USA, Canada, the Netherlands, Singapore, China, Spain, and Argentina. The core design compared lead-time TTO to discrete choice modeling [14]. Each of the country studies tested different aspects of the core protocol.

### Sample

The sample was drawn from the general population of the city of Buenos Aires and its metropolitan area, covering individuals between 18 and 80 years of age. The selection of participants was entrusted to IPSOS, a private survey consultancy specialized in social and marketing research. The pilot was held in two locations, Buenos Aires City and Lomas de Zamora City (in the metropolitan area) between 30 May and 3 June 2011. In order to assure the representativeness of the sample [15], quota sampling by gender, age, and socioeconomic status was performed. Data were collected in group interviews using a digital aid.

### Design

Interview scripts and a digital aid were developed to present each variant of the TTO. As shown in Fig. 1, Options A and B were displayed horizontally using the same colors to represent a particular state (green for full health and blue to represent H*i*). The iterative process used to reach the point of indifference was automated within the EQ-VT digital aid [14]. Additionally, the EQ-VT captured all participants’ responses to the task and the amount of time they needed to complete it.

### Response tasks

The tasks, instructions, follow-up, and feedback questions comply with standard procedures for translation and back-translation from English to Argentinean Spanish. The IECS researchers (VI, LRA) attended two training workshops in the Netherlands in order to standardize the interview protocol. They replicated this protocol in three workshops in Buenos Aires with two other IECS researchers (FA, NE) and the IPSOS team of interviewers.

The protocol replicated the one that was used in the core multinational pilot study, which included lead-time TTO, a discrete choice (DC) model (paired comparisons), and the visual analog scale [14]. The informed consent form was adapted and approved by the Hospital Italiano de Buenos Aires Institutional Review Board (IRB) in May 2011.

The study used a split-sample design, whereby a random half of the sample would get lead-time TTO and the other half lag-time TTO. All the tasks were administered using EQ-VT. The interview began with a warm-up exercise of self-rated health, followed by ten paired comparisons per participant. The pairs in the DC task were selected on the basis of a blocked design with 20 blocks of 10 pairs (total number of states evaluated = 400). The DC task was followed by TTO valuations of five EQ-5D-5L states (total number of states evaluated by TTO = 10).

To conduct the Argentinean arm of the multinational study, we selected one variant of lead-time TTO and one of lag-time TTO, with a duration of 5 years for the health state to be evaluated and 10 years of full health (2:1 ratio). This ratio was selected in light of the results of a previous study conducted by Devlin et al. in the UK [11]. For the TTO task, respondents were randomly assigned to one of two fixed blocks of states, each composed of mild, moderate, and severe health states. They were then instructed to use either a lead-time TTO or a lag-time TTO method.

Both the DC and the TTO tasks were preceded by a brief animation sequence designed to explain and illustrate what they were supposed to do. Upon completion of the task, the participants were asked a series of follow-up questions. They were asked to evaluate the difficulty and their understanding of the tasks, to give some background information, and to answer some structured feedback questions.

### Analysis

For TTO responses, the mean, median, standard deviation, and interquartile range (IQR) were calculated. To analyze the difference between lead-time TTO and lag-time TTO groups, we performed a *t* test. The primary comparison variable comprised the values obtained through the two TTO methods and its differences. These two variants were also compared in other aspects: (1) procedural aspects (time spent by the respondents on each task, number of steps taken to reach the point of indifference for each health state; number of times the respondent reset the protocol and how understandable it was; proportion of subjects that took a different number of steps before reaching the point of indifference); (2) logical inconsistencies between pairs of health states in each of the blocks; (3) the relationship between the health-state values and the severity of the valued states. Severity is measured using a ‘misery index,’ an additive representation of the levels of the five dimensions. The misery index for the EQ-5D-5L ranges from 1 + 1 + 1 + 1 + 1 = 5 (for the state of full health, 11111) to 5 + 5 + 5 + 5 + 5 = 25 (for the worst health state defined by the EQ-5D-5L descriptive system, 55555). All statistical analyses were conducted using STATA^®^ MP 9.2.

## Results

A total of 414 participants between 18 and 80 years old were interviewed. Twenty-seven subjects were excluded from the analysis because they had given the same value to all five health states presented. Also, 17 tasks that had been performed in less than a second were excluded under the premise that the task had not been understood. The lead-time variant was performed by 209 subjects, while 178 did the lag-time variant. There were no significant differences between the groups regarding baseline characteristics and their self-reported state of health, nor between the 27 excluded participants and the rest of the sample (see Table 1). Subjects’ characteristics were similar to those of the general population of Argentina, except for the subjects’ higher educational level [15].

**Table 1:** Sample characteristics (demographics) and EQ-5D-5L responses

	Lead-time TTO	Lag-time TTO	*p* (*t* test/*χ* ^2^)
Number of participants (%)	209 (52)	178 (48)	
Age (years) mean/SD	39.2/13.7	38.9/14.4	0.81
Female *n* (%)	107 (51.2)	94 (52.8)	0.75
Educational level achieved (high school or higher)	92.8 %	88.8 %	0.17
*EQ-5D-5L*			
No mobility problems	88.5 %	91.6 %	0.32
No self-care problems	96.2 %	97.2 %	0.58
No limitation of usual activities	88 %	92.1 %	0.18
No pain or discomfort	60.8 %	68.5 %	0.11
No anxiety or depression	64.1 %	71.9 %	0.1
Self-reported health (VAS) mean/SD median/IQR	84.4/12.5	85.5/11.7	0.4
	86/80–93	90/80–92	

**Fig. 2:**
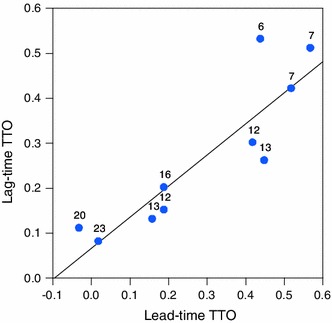
Mean health-state values by state and TTO variant

No significant differences were found regarding the values obtained in both groups for all the evaluated states, except for a statistically significant difference of 0.19 in state 33133 (*p* = 0.04). Table 2 shows the mean and median values for each of the states. The scatter graph shows the amount of agreement between lead-time and lag-time TTO means (see Fig. 2). Values for lead-time were higher than those for lag-time for states of intermediate severity, while the reverse was the case for the extremes (both the mild and the severe states).

**Table 2:** Health-state values by TTO variant

State	Lead-time TTO			Lag-time TTO			*t* test (mean)	
	*n*	Mean (SD)	Median (IQR)	*n*	Mean (SD)	Median (IQR)	*p* value	Diff (CI 95 %)
21111	109	0.44 (0.59)	0.6 (0, 0.95)	85	0.53 (0.51)	0.7 (0, 1)	0.27	−0.09 (−0.25; 0.07)
12112	99	0.52 (0.50)	0.6 (0.3, 1)	92	0.42 (0.65)	0.58 (0, 0.9)	0.22	0.1 (−0.06; 0.27)
11221	107	0.57 (0.49)	0.7 (0.4, 1)	83	0.51 (0.53)	0.7 (0.3, 1)	0.38	0.07 (−0.08; 0.21)
52221	100	0.19 (0.68)	0.38 (0, 0.6)	91	0.15 (0.69)	0.3 (−0.2, 0.6)	0.71	0.04 (−0.16; 0.23)
11145	109	0.42 (0.56)	0.5 (0, 0.95)	85	0.3 (0.61)	0.4 (0, 0.9)	0.15	0.12 (−0.04; 0.29)
33133	100	0.45 (0.58)	0.5 (0, 1)	90	0.26 (0.64)	0.48 (0, 0.8)	0.04	0.19 (0.01; 0.36)
44113	99	0.16 (0.62)	0 (−1, 0.6)	90	0.13 (0.72)	0.1 (−0.2, 0.75)	0.75	0.03 (−0.16; 0.22)
52324	108	0.19 (0.67)	0.1 (0, 0.7)	85	0.2 (0.70)	0.4 (0, 0.8)	0.9	−0.01 (−0.21; 0.18)
55523	107	−0.03 (0.54)	0 (−0.6, 0.5)	82	0.11 (0.75)	0.05 (−0.1; 0.7)	0.2	−0.14 (−0.35; 0.08)
53555	100	0.02 (0.76)	0 (−0.25, 0.5)	92	0.08 (0.76)	0.15 (−0.4, 0.6)	0.57	−0.06 (−0.28; 0.15)

The mean observed values per EQ-5D-5L state for lead-time and lag-time TTO are shown in Fig. 4 in the "Appendix". The values ranged from 0.53 for 21111 to 0.08 for 53555 for lag-time TTO and between 0.44 and 0.02 for lead-time TTO.

**Fig. 3:**
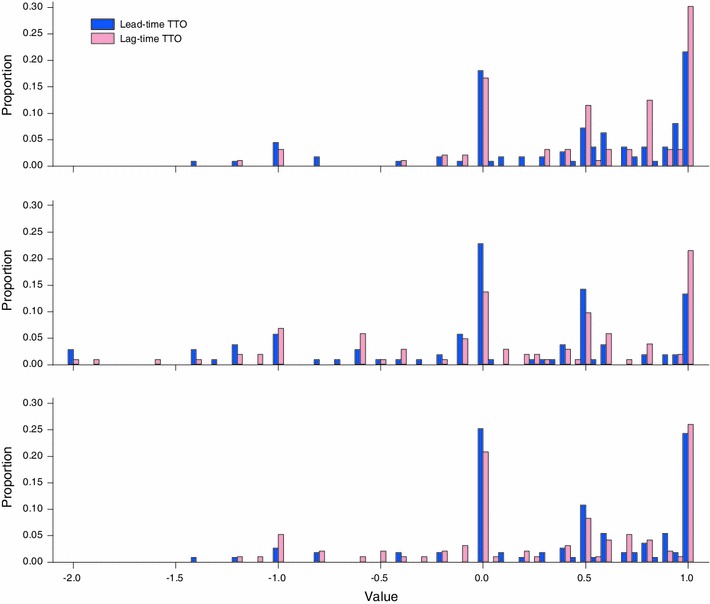
Valuation distribution for three health states: mild (*upper graph*), moderate (*lower graph*) and severe (*middle graph*)

**Fig. 4:**
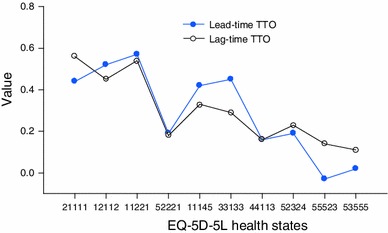
Mean observed values per EQ-5D-5L state for lead and lag-time TTO

Marked peaks around zero were observed in all the valued states, as shown in Fig. 3. A significant number of respondents valued the states presented as similar to being dead (365 responses). Between 60 and 70 percent of them took only two steps to make this choice. In search of an explanation for this phenomenon, the presence of an order effect was explored. We found a greater percentage of responses *U* = 0 in the last state valued compared with the first (27.1 vs. 16.2 percent in lead-time TTO and 23.32 vs. 13.71 percent in lag-time TTO). Regarding the percentage of non-trading responses (where poor health is valued at 1), we found no difference between lead-time and lag-time TTO (19.65 vs. 19.54 percent, *p* = 0.95).

From each block of five states that the participants valued, at least two of the states could be logically ordered (i.e., 44223 vs. 12112). The extent to which there was logical consistency was compared between the lead-time TTO and lag-time TTO groups. There were no differences regarding consistency between the blocks or TTO variants. The level of inconsistency was high (41.2 % in block 1 and 46.9 % in block 2).

A subgroup analysis was carried out, excluding the states in which the values were assessed in two steps. The goal of the analysis was to avoid potential bias. No major differences were found between lead-time TTO and lag-time TTO. The difference found in the original analysis for state 33133 disappeared, while a new one emerged for state 11145. The mean difference was 0.24 (*p* = 0.02).

There were no significant differences between the lead-time and lag-time TTO groups in their responses to the follow-up questions, nor did these groups differ in the way the participants responded to the tasks (the amount of time they needed to value each health state, the number of steps they took to reach indifference in the TTO tasks, etc.). Ninety percent of the participants found the instructions clear and easy to understand, while half had some difficulty deciding on their answer. It is noteworthy that just over half of the participants indicated that when valuing the poor health states, they took into account the possibility that a new treatment or relief would be available. More details on this perspective are provided in the "Appendix".

## Discussion

Lead-time and lag-time TTO are relatively new variants that were developed to address problems with conventional TTO. The EuroQol Group chose to incorporate the lead-time TTO in their EQ-5D-5L pilot valuation [14] study in an effort to overcome the known issues. As one of the sub-studies of the multinational EQ-5D-5L pilot study, we compared the two TTO variants (lead-time and lag-time TTO) in order to determine whether there were differences between them.

There are few studies in the literature comparing these variants; most focus on lead-time TTO exclusively [7, 11, 12], and none used the EQ-5D-5L instrument. One study [11], which included three variants of lead-time TTO and one of lag-time TTO, found that none of the variants had either a systematically higher or lower proportion of values >0 across all states. The lag-time variant gave considerably higher values for the severe states compared to its lead-time counterpart. This difference was attributed purely to the positioning of poor health first and full health later. The lag-time variant also completely eliminated ‘non-trading’ responses; even in very mild health states, all participants were willing to trade at least some time.

Our pilot was conducted in a sample from the general population of Argentina. The analysis focused on testing for potential differences between the lead-time and lag-time TTO, in terms of both the EQ-5D-5L values produced and various measures of the ‘process’ by which participants arrived at those values. This was a randomized study, and the population characteristics were similar across the groups, making comparisons between them more valid. Minor imbalances in the number of subjects assigned to each group—arising from technical problems with the EQ-VT in the initial phase of recruitment—have no bearing on the results and interpretation of the study.

We found no relevant differences in the health-state values generated by the two methods or in the process (number of steps taken by participants to reach the point of indifference and the amount of time to complete the protocol). Some of the states had higher values in the lag-time TTO group, while the opposite was true for other states. Only one state was found to be statistically different between the two variants.

An unexpected observation made in both lead-time and lag-time TTO—and something that has also been observed in all countries in the multinational pilot study using the lead-time TTO variant—was that a substantial peak occurred around 0 in all health states. This peak probably explains the low mean values produced for mild states in this study, but also the high level of inconsistency. This finding—the peak in values at 0—has not been previously reported in studies using lead-time TTO [3, 11, 12]. Therefore, a likely explanation would lie in the interview protocol for this study: it used a digital aid in a group setting without personalized assistance (there was one interviewer for every ten participants who were being interviewed at the same time). The time to complete the task was also unexpectedly short, suggesting that the participants may not have had adequate concentration and could have rushed to complete the task. This conclusion is also suggested by the EQ-VT iteration process. In that process, for all states, the second step in the TTO tasks involved (as a ping-pong technique) going to a value of 0; from there, the participants had the choice of declaring indifference (in which case their value was recorded as 0) or moving between options above or below a value of 0. The large proportion choosing a value of 0 after only two steps suggests that participants were tempted to finish the task earlier. They then reached the indifference point without thinking about the fact that they were assigning the health state a value of ‘as bad as being dead.’ These results should thus be interpreted bearing this fact in mind.

In conclusion, we did not find any meaningful or systematic differences between the lead-time and lag-time TTO variants. There are two alternative explanations for our findings: (1) the results are eclipsed by the nature of the group interview protocol, which produced a high number of states valued as zero; or (2) both variants are equivalent. In order to eliminate the potential noise introduced by the interview protocol, future research should administer TTO tasks in face-to-face interview settings. So doing would promote the engagement of the participants and thereby be conducive to a valid set of preference data.

## Appendix

See Tables 3, 4, 5 and Fig. 4.

**Table 3:** Blocks of states

	State no.	MO	SC	UA	PD	AD
Block 1	1	1	2	1	1	2
	2	5	2	2	2	1
	3	3	3	1	3	3
	4	4	4	1	1	3
	5	5	3	5	5	5
Block 2	6	2	1	1	1	1
	7	1	1	2	2	1
	8	5	2	3	2	4
	9	5	5	5	2	3
	10	1	1	1	4	5

**Table 4:** Cognitive debriefing: different levels of agreement, percentage of people who agreed (strongly agree or agree)

Questions	Lead-time TTO (%)	Lag-time TTO (%)
Instructions were clear	89.5	92.2
Questions were easy to understand	90.0	90.5
It was difficult to decide the answer	49.0	50.6
The difference between the lives presented was easy to tell	82.8	83.8
The time they asked to imagine was too long	32.7	34.5
A new treatment or relief was possible	55.3	56.5
I will get used to living with impairment	50.5	46.4
The most important thing is if I would able to work	72.6	66.1
Difficult to imagine the health states	56.8	53.1

**Table 5:** Respondents’ process in performing the task (per each health state evaluated)

		Lead-time TTO	Lag-time TTO
No. of resets^∞^	0	94.5 %	94.9 %
	1	4.4 %	4.5 %
	2	1 %	0.5 %
	3	0.1 %	0.1 %
No. of moves^∫^	Mean	7.2	7
	SD	8.8	8.5
	Range	1–103	1–103
	Median	5	5
Time (s)^∆^	Mean	53.2	55
	SD	70.2	72.8
	Median	28.9	29.2
	IQR	14.1–61.8	13.9–66.7
Less than 1 min (%)^∂^		73.9 %	72.2 %

^∞^Number of times participants reset the valuation of one health state

^∫^Number of clicks made by the participant to value one health state (1 click was necessary to select the indifference point by clicking the button “A&B are the same”)

^∆^Time in seconds needed to complete the valuation of one health state

^∂^Participants (%) who used less than a minute to value one health state
